# 

**Published:** 1904-01-30

**Authors:** 


					THE HOSPITAL. "The standard of highest purity."?Lancet. January 30th, 1904,
CADBURY'S U CADBURY'S
COCOA jSL Ja^ n COCOA xt'st'h*
'?A PERFECT FOOD." NO DRUGS OR ALKALIES USED* As? V ^
scoir Westejui Infirmary
No. 905, Vol. XXXV. [^fewlpfper!] Saturday, Jan. 30th, 1904. [
see p. 310. ] PRICE THREEPENCE.

				

